# Validation of protein models by a neural network approach

**DOI:** 10.1186/1471-2105-9-66

**Published:** 2008-01-29

**Authors:** Paolo Mereghetti, Maria Luisa Ganadu, Elena Papaleo, Piercarlo Fantucci, Luca De Gioia

**Affiliations:** 1 Department of Chemistry, University of Sassari, Via Vienna 2, 07100, Sassari, Italy; 2 Department of Biotechnology and Biosciences, University of Milano-Bicocca, P.za della Scienza 2, 20126, Milan, Italy

## Abstract

**Background:**

The development and improvement of reliable computational methods designed to evaluate the quality of protein models is relevant in the context of protein structure refinement, which has been recently identified as one of the bottlenecks limiting the quality and usefulness of protein structure prediction.

**Results:**

In this contribution, we present a computational method (Artificial Intelligence Decoys Evaluator: AIDE) which is able to consistently discriminate between correct and incorrect protein models. In particular, the method is based on neural networks that use as input 15 structural parameters, which include energy, solvent accessible surface, hydrophobic contacts and secondary structure content. The results obtained with AIDE on a set of decoy structures were evaluated using statistical indicators such as Pearson correlation coefficients, Z_*nat*_, fraction enrichment, as well as ROC plots. It turned out that AIDE performances are comparable and often complementary to available state-of-the-art learning-based methods.

**Conclusion:**

In light of the results obtained with AIDE, as well as its comparison with available learning-based methods, it can be concluded that AIDE can be successfully used to evaluate the quality of protein structures. The use of AIDE in combination with other evaluation tools is expected to further enhance protein refinement efforts.

## Background

The very large and continuously increasing amount of data obtained by genome sequencing makes the development of reliable computational methods capable to infer protein structures from sequences a crucial step for functional annotation of proteins. In fact, functional annotation is often strictly dependent on the availability of structural data, which in turn are still difficult to obtain experimentally. As a consequence, efforts and progresses in high throughput X-ray and NMR methods need to be accompanied by computational techniques suitable for three-dimensional structure predictions, such as homology modeling, fold recognition or ab-initio methods [1-7], which are intrinsically characterized by different levels of accuracy.

In parallel to the development and improvement of prediction methods, reliable and accurate evaluation tools are necessary to check the quality of computational protein models [8,9]. Moreover, in the context of protein structure refinement, which has been recently identified as one of the bottlenecks limiting the quality and usefulness of protein structure prediction [1], it has been noted that improvements in the selection of the most native-like model from an ensemble of closely related alternative conformations can be crucial. The increasing importance of the field of quality assessment methods is demonstrated by the introduction of a dedicated section in the latest CASP edition (CASP7) [10].

To evaluate protein structures, several different scoring functions have been developed, which can be classified into different categories depending on the principles and on the structural features considered in the evaluation. Physical scoring (energy) functions aim to describe the physics of the interaction between atoms in a protein and are generally parameterized on molecular systems smaller than proteins [11]. Knowledge-based scoring functions are designed by evaluating the differences between some selected features of a random protein model and the characteristics of a real protein structure [12-16].

Learning-based functions can be developed by training algorithms to discriminate between correct and incorrect models [17]. Independently by the category, scoring functions are generally tested by examining their capability to detect the native structure among a set of decoys [18], which can be generated in several different ways [19-21].

It is important to note that the performance of learning-based functions are generally strongly dependent on the specific aim for which they were developed, and consequently on the training set used. As an example, ProQ, a neural network based method developed to predict the quality of protein models [17], was specifically designed to discriminate between correct and wrong models, i.e. to recognize folds that are not compatible with a protein sequence. In fact, ProQ was recently combined successfully with the Pcons [22] fold recognition predictor and ranked as one the best methods in a recent survey of quality assessment methods [10]. Other reliable and extensively used computational methods used to validate the quality of protein structures are PROSA [23], ERRAT [24], Verify 3D [25,26], PROCHECK [27], what-if [28], PROVE [29] and victor/FRST [15].

In the present contribution, we present a computational method (Artificial Intelligence Decoys Evaluator: AIDE) that is able to reliably and consistently discriminate between correct and incorrect protein models. In particular, the quality of the protein structure is evaluated with neural networks using as input 15 structural parameters, which include solvent accessible surface, hydrophobic contacts and secondary structure content. In the first section of the paper, the neural network structure and the training procedure are presented and discussed. In the second section, the performance of the neural network is evaluated, compared to available methods, and critically discussed.

## Results and Discussion

The evaluation of the quality of protein structures is generally carried out calculating a score which is a function of a set of parameter values computed for the protein model under study. In our computational procedure, the description of the relation between the parameters space and the scoring values is obtained using neural networks, because of their ability to describe complex non-linear relationships among data.

### Selection of protein parameters related to structure quality

Among the possible parameters that can be computed for a protein structure, we have selected some properties that are expected to be related to structure quality: solvent accessible surface of hydrophobic and hydrophilic residues, secondary structure content, the fraction of secondary structure content of the model fitting with that predicted by PSIPRED [30], number of hydrophobic contacts, and selected PROCHECK parameters [27] (see Methods for details). It should be noted that other possibly relevant parameters, such as the number of hydrogen bonds, have not been used due to intrinsic difficulties in the normalisation of their values.

### Selection of the parameters used to evaluate structure similarity

A key issue for evaluating the quality of a predicted protein structure is the measure of its "distance" relative to the "real" structure, experimentally obtained by X-ray diffraction or NMR. Since AIDE has been developed to evaluate protein models that are often characterized by the correct fold but may differ for local details, the backbone root mean square deviation (RMSD) of the protein model relative to the X-ray structure can be considered a suitable measure of structure similarity [31]. In fact, it is well known that the proper evaluation of the quality of protein structures can be a non-trivial task, often depending on the methods used to generate protein models. Therefore, several other measures of protein structure similarity have been formulated, the most commonly used being: GDT-TS [32], LG-score [32], TM-score [33] and MaxSub [34], which have also been adopted in the present work.

### Selection and optimization of the neural networks

A preliminary evaluation of the relative importance of each parameter in the description of structure quality was obtained using a linear models built with the M5-prime attribute selection algorithm [35], as implemented in Weka 3.4.2 [36]. A different linear model was computed for each accuracy measure. Analysis of the linear models revealed that the secondary structure content and the solvent accessible surface have the highest importance in all models. Moreover, results show that it is not possible to exclude any parameter since non negligible weights are associated to all selected parameters, when the accuracy measures chosen are considered as a whole (Additional File 1).

The neural networks forming the core of AIDE are four layers feed forward neural networks with fifteen neurons (corresponding to the selected parameters) in the input layer, two hidden layers formed by two neurons each, and one neuron in the output layer. A linear activation function was chosen for all neurons. Indeed, different combinations of hidden layers (one or two) and different numbers of hidden neurons per layer (from two to ten nodes per layer) were tested. In addition, we tested also different activation functions of the neurons (sigmoid, log-sigmoid and linear functions). It turned out that, among the different combinations, the neural network featuring two hidden layers formed by two neurons gave the best results. In fact, an increase in the number of neurons led to poorer performances, probably due to the increased difficulties in the optimization procedure arising from the augmented network complexity. To carry out the optimization of neural networks, we have implemented the attractive-repulsive particle swarm optimization algorithm (AR-PSO) [37], as explained in Methods. Training of the neural networks using more conventional approaches (Gradient descent, Levenberg-Marquardt), led to slightly lower performances (Additional file 2). This may be due to the greater exploration ability that characterize the PSO methods.

AIDE was trained and tested on datasets of all-atoms protein decoys for which the three-dimensional structures are available. Since it is known that methods used for building decoys may introduce some systematic bias, it is important to benchmark a scoring function on different decoy sets in order to assess its generality. The overall dataset used in the present study is composed by an ensemble of widely used all-atom datasets containing models of different proteins (4state-reduced, fisa, fisa-casp3, rosetta all-atoms, CASP5, CASP7, Livebench2, lmds, and hg-structal [19-21,38-40]), plus a molecular dynamics set that was generated in our laboratory from X-ray structures (see Methods).

After computation of the structural parameters to be inserted in the neural networks, the overall dataset was subdivided into a training and a test set, which were composed by 13693 and 49126 structures, respectively. The training-set includes only the proteins belonging to the LiveBench2 and CASP7 decoy sets (13693 model structures built on 96 different proteins). The test-set includes the lmds, CASP5, hg_structal, MD, Rosetta and 4state-reduced subsets (49126 models build on 97 proteins). The LiveBench2 and CASP7 decoy sets were chosen as training sets because they contain models build with different methods and of different protein size, ranging from 20 to 500 residues. No protein contained in the training set is present also in the test set.

Then, a population of 50 neural networks was trained starting from different initializations of the structural parameters. The network featuring the best performance (the highest correlation coefficient on the training set) was selected as the working network in AIDE.

A different neural network was trained for each measure of structure similarity chosen to evaluate proteins quality (RMSD, TM-score, GDT-TS, LG-score and MaxSub). Therefore, five different versions of AIDE were obtained from the training procedure, referred to in the following as AIDE RMSD, AIDE TM-score, AIDE GDT-TS, AIDE LG-score and AIDE MaxSub.

### Assessment of AIDE performance

The performances of the different version of AIDE have been compared to results obtained from widely used methods developed to evaluate protein models quality.

The performances of the different methods were evaluated using a test-set which includes lmds, CASP5, hg_structal, MD, Rosetta and 4state-reduced subsets. The LiveBench2 and CASP7 sets were already used for training AIDE and therefore were not used in the comparative evaluation.

The Pearson correlation coefficient, *Z*_*nat *_and fraction enrichment (F.E.), which give indications about a method ability to assign good scores to good models, have been computed and results are collected in Tables 1, 2, 3.

**Table 1:** Pearson correlation coefficients. For each dataset belonging to the test-set the Pearson correlation coefficient between the predicted and the computed values is reported. The performance of AIDE is compared to that of ProQ and Victor/FRST validation softwares.

	lmds	4state_reduced	CASP5	fisa	MD	hg_structal	ROSETTA	average
AIDE RMSD	0.39	0.42	0.15	0.63	0.61	0.69	0.27	0.45
AIDE TM-score	0.39	0.32	0.38	0.48	0.89	0.70	0.43	0.51
AIDE GDT-TS	0.45	0.34	0.28	0.58	0.77	0.73	0.44	0.51
AIDE LG-score	0.52	0.31	0.22	0.29	0.77	0.48	0.38	0.42
AIDE MaxSub	0.39	0.34	0.36	0.55	0.73	0.70	0.40	0.49
ProQ LG-score	0.20	0.62	0.48	0.18	0.81	0.80	0.06	0.45
ProQ MaxSub	0.15	0.48	0.39	0.14	0.77	0.76	0.05	0.39
Victor GDT-TS	-0.29	-0.53	-0.29	-0.05	-0.78	-0.75	-0.23	-0.41

**Table 2:** 10%-fraction enrichment. The 10%-fraction enrichment is shown for each dataset belonging to the test-set. The performance of AIDE is compared to that of ProQ and Victor/FRST validation softwares.

	lmds	4state_reduced	CASP5	fisa	MD	hg_structal	ROSETTA	average
AIDE RMSD	15.20	42.58	37.10	25.00	48.19	43.67	20.80	33.22
AIDE TM-score	1.84	31.18	34.84	19.50	72.29	44.82	26.49	32.99
AIDE GDT-TS	2.07	32.68	29.86	11.50	72.28	50.57	25.29	32.03
AIDE LG-score	25.80	34.40	31.67	17.52	67.47	42.53	29.96	35.62
AIDE MaxSub	3.22	33.54	35.29	10.00	73.49	44.82	24.12	32.06
ProQ LG-score	18.30	54.78	39.59	12.50	72.45	74.71	13.30	40.80
ProQ MaxSub	1.95	52.84	45.24	12.00	65.86	67.84	43.69	41.34
Victor GDT-TS	14.40	42.57	28.50	4.0	63.85	54.02	11.61	31.27

**Table 3:** *Z*_*nat*_. Comparison of *Z*_*nat *_values obtained using AIDE and other protein structure validation softwares. ProQ values have been obtained from Ref. 17.

	lmds	4state_reduced
AIDE RMSD	2.4	0.5
AIDE TM-score	3.4	2.9
AIDE GDT-TS	3.5	3.1
AIDE LG-score	2.0	1.6
AIDE MaxSub	3.1	3.1
ProQ LG-score	3.9	4.4
ProQ MaxSub	1.8	3.5
Victor GDT-TS	3.5	4.4
Errat	3.1	2.5
Prosa II	2.5	2.7
Verify 3D	1.4	2.6

Analysis of Pearson correlation coefficients (Table 1) shows that, according to this statistical indicator, the different AIDE versions behave quite similarly. Most importantly, average AIDE performances are similar or slightly better than those obtained by two state-of-the-art methods such as ProQ [17] and Victor [15]. It is also noteworthy that the performance of AIDE changes significantly moving through the different subsets forming the test-set. In particular, very high correlation coefficients are obtained with the MD and hg_structal datasets (correlation coefficient in the range 0.61–0.89 and 0.48–0.73, respectively), whereas low values of Pearson coefficients are associated to the CASP5 dataset (0.15–0.38). Relatively different values of Pearson correlation coefficients are obtained also with ProQ and Victor. In particular, and differently from AIDE, low correlation coefficients are obtained by ProQ for the Rosetta subset, and by Victor for the fisa subset (Table 1). The factors responsible for such non-homogeneous performances of the methods, when applied to different datasets, could not be unrevealed and might require further dissection of the test-set. In light of these results and observations it can be concluded that, even if the overall performances of AIDE, ProQ and Victor are similar, these methods can behave very differently on protein models obtained using different approaches, suggesting that the combined use of AIDE, ProQ and Victor could be useful to properly evaluate the quality of a protein structure.

Analysis of F. E. values (Table 2) shows again quite similar overall performances of AIDE, ProQ and Victor. However, the average F. E. values obtained using ProQ are consistently higher (by 5–10%) relative to the corresponding values obtained with Victor and AIDE. A more detailed analysis of F. E. values obtained from the different subsets composing the test set highlights some interesting trends. F. E. values obtained from the lmds and fisa subsets are consistently lower than the average. Moreover, AIDE and ProQ versions trained using different parameters to evaluate structure similarity can give quite different results. The latter observation is particularly evident for the lmds subset. It is also interesting to note that the best performances on the different subsets forming the test set are often obtained by different methods. As an example, the best F. E. values for the fisa subset are obtained using AIDE, whereas the best values for the hg_structal subset are obtained with ProQ, further suggesting that the combined use of the different methods can be a good strategy to obtain a more confident evaluation of the quality of a protein structure. *Z*_*nat *_allows to evaluate how (and if) the different methods distinguish the native (X-ray) structure from the ensemble of its models (Table 3). In this case it was possible to extend the comparison to other methods widely used to evaluate protein structures quality (Errat, Prosa II and Verify 3D). Only the lmds and 4state_reduced subsets have been used in this comparison because these are the only datasets in common among all the compared methods for which data are available. Analysis of *Z*_*nat *_values reveals that ProQ and Victor have better performances in this statistical test, whereas AIDE results are generally comparable to those obtained with Errat, Prosa II and Verify 3D. Notably, very low *Z*_*nat *_scores are obtained using AIDE RMDS and AIDE LG-score on the 4state_reduced subset.

**Figure 1:**
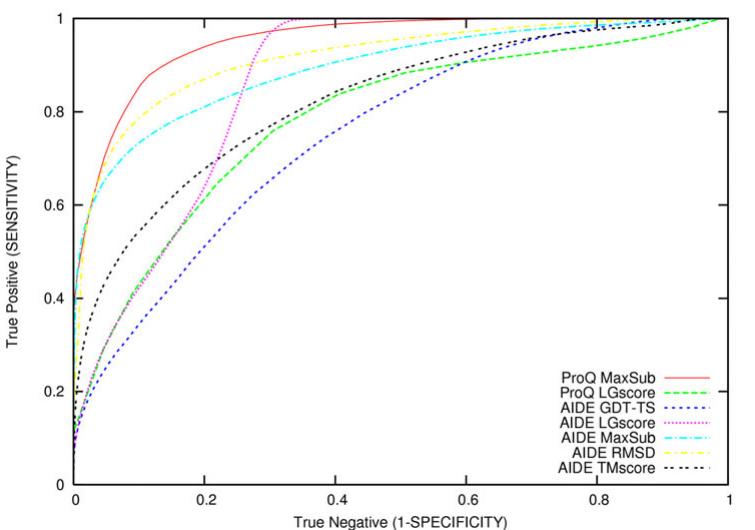
**ROC plots**. Comparison of AIDE and ProQ as obtained using a receiver operating characteristic (ROC) plot computed on the complete test-set (lmds, CASP5, hg_structal, MD, Rosetta and 4state-reduced datasets). Each line represents the ROC curve obtained with a specific AIDE or ProQ version. The ProQ MaxSub and the ProQ LG-score are plotted as solid:red and dashed:green line, respectively. The different AIDE versions are plotted as follow: GDT_TS dashed:blue; LG-score dotted:purple; MaxSub dashed-dotted:cyan; RMSD dashed-dotted:yellow; TM-score dashed:black.

It should be noted that *Z*_*nat *_and F.E. do not give information about the ability of a method to assign low scores to bad models, i. e. these statistical indicators do not allow to check if a method is confusing different classes. To explore this issue we have qualitatively compared AIDE and ProQ performances, superposing the ROC plots (see Methods) computed on the test-set for each different performance function (Figure 1). According to this analysis, ProQ MaxSub exhibits the greatest overall accuracy, whereas AIDE GDT-TS has the lowest accuracy.

Considering the different AIDE versions, a clear distinction can be observed when comparing the overall accuracy of AIDE RMSD and AIDE MaxSub relative to AIDE LGscore, AIDE GDT-TS and AIDE TMscore (Figure 1). Notably, a similar difference was not evident when considering the correlation coefficients or the fraction enrichment test. It is also important to note that AIDE LGscore behaves very similarly to ProQ LGscore until about 60% of sensitivity, whereas at higher sensitivity levels AIDE outperforms ProQ LGscore. These observations further corroborate the hypothesis that the combined use of ProQ and AIDE should give improved results in the evaluation of the quality of three-dimensional protein models.

### The web interface of AIDE

The availability of five different AIDE versions gives a nice picture of the overall performance of the method. However, the overloading of output information can become a drawback for the user interested only in the most relevant results. In fact, the analysis of AIDE performance has shown that the five different versions of AIDE are generally characterised by similar behaviour (see Table 1, 2, 3). To better evaluate the degree of correlation among different AIDE versions we have carried out a principal component analysis on the Pearson correlation matrix of the descriptors chosen to evaluate models quality. This analysis reveals a strong correlation between TM-score, GDT-TS and MaxSub. The different clustering of TM-score, GDT-TS and MaxSub relative to RMSD and LG-score is mainly due to the inverse relationship between the two families(Additional files 3 and 4). Therefore, two (GDT-TS and the MaxSub) of these highly correlated parameters have been excluded from the output of the AIDE program available on the Internet [41]. Moreover, to help the user in the evaluation of AIDE results, we have defined a threshold for each predicted parameter, in order to discriminate between incorrect and correct models. In particular, correct models should have TM-score ≥ 0.31, RMSD ≤ 4.96 Å and LG-score ≤ 0.35. These thresholds were chosen using a dataset of manually assessed models composed by some CASP5 targets belonging to the new fold and fold recognition categories. According to the visual evaluation of Aloy and coworkers [42], the models were divided into three class: class 2 ("excellent") when the overall fold is correct, class 1 ("good") when the model is considered partway to the correct fold, and class 0 for all the other models. For each model, the TM-score, LG-score and RMSD were computed (Additional files 5, 6, 7) and the average value for the models belonging to the "excellent" class was used as threshold. To further evaluate the classification ability using the chosen thresholds, the sensitivity and the specificity based on the ROC plots were also computed (Additional file 8).

## Conclusion

In this paper we have presented AIDE, a neural network system which is able to evaluate the quality of protein structures obtained by prediction methods.

AIDE differs from other evaluation methods mainly for : i) a different choice of the parameters used to describe the protein structure, ii) a different choice of the parameters related to structure quality, iii) a novel strategy used to optimize the neural networks. AIDE overall performances are comparable to recently published state of the art methods, such as ProQ [17] and Victor [15]. However, detailed comparative analysis of results obtained using AIDE, ProQ and Victor reveals that the three methods have different and often complementary ability to properly assess the quality of protein structures. This observation suggests that the combined use of AIDE, ProQ and Victor could increase the reliability in the evaluation of protein structures quality. AIDE is presently available on the Internet [41].

## Methods

### Protein datasets

The 4state-reduced set is an all-atom version of the models generated by Park & Levitt [19] using a four-state off-lattice model.

The fisa and fisa-casp3 sets contain decoys of four small alpha-helix proteins. In these sets main chains were generated using a procedure of fragment insertion based on simulated annealing: native-like structures were assembled from a combination of fragments of known unrelated protein structures characterized by similar local sequences, using Bayesian scoring functions [20]. The side chains of fisa and fisa-casp3 were modeled with the software package SCWRL [43].

The hg-structal is a set of hemoglobin models generated by homology modelling.

The lmds subset [19,44] was produced by Keasar and Levitt by geometry optimizations carried out using a complex potential that contains a pairwise component, as well as cooperative hydrogen bonds terms. The Rosetta all-atom decoys were generate with the ROSETTA method developed by David Baker [21]. The molecular dynamics set of decoys was generated by molecular dynamics (MD) simulations carried out in vacuum with the software GROMACS 3.2 [45,46]. Each protein structure was submitted to 100 ps of simulation using the OPLS force field [47]. MD simulations were performed in the NVT ensemble at 600 K, using an external bath with a coupling constant of 0.1 ps [48]. The LINCS algorithm [49] was adopted to constrain bond lengths of heavy atoms, allowing us to use a 2 fs time step. Van der Waals and Coulomb interactions were truncated at 8 Å, while long-range electrostatics interactions were evaluated using the particle mesh ewald summation scheme [50]. The Van der Waals radii were increased to 4 Å for all atoms, in order to speed up the unfolding process [51].

Snapshots from the trajectory have been extracted every 0.4 ps, collecting 250 misfolded structures for each protein, with a backbone RMSD (root mean square deviation between the initial structure and each snapshot) ranging from 0 to about 10 Å.

In addition to these decoy datasets, the CASP5 [38], CASP7 [39] and LiveBench2 [40] sets were also included.

The complete dataset contains 62819 protein models build on 193 proteins.

### Training-set and test-set

The dataset was splitted into two disjoint sets : a training-set and a test-set. The training-set includes only proteins belonging to the LiveBench2 and CASP7 decoys sets (13693 model structures built on 96 different proteins). The test-set includes the lmds, CASP5, hg_structal, MD, Rosetta and 4state-reduced datasets (49126 models build on 97 proteins).

### Parameters-Descriptors used in the neural network

The relative solvent accessible surface (*rSAS*) was computed as follow:

$${\mathrm{rSAS}}_{\mathrm{hydrophobic}}=\frac{{\mathrm{SAS}}_{\mathrm{hydrophobic}}}{{\mathrm{SAS}}_{\mathrm{total}}}$$

$${\mathrm{rSAS}}_{\mathrm{hydrophobic}}=\frac{{\mathrm{SAS}}_{\mathrm{hydrophobic}}}{{\mathrm{SAS}}_{\mathrm{total}}}$$

where the residues A, L, V, I, P, F, M, W were considered as hydrophobic and the *SAS*_*total *_is the total solvent accessible surface computed using NACCESS [52].

The secondary structure was evaluated with the DSSP program [53], in which the typical 8-state DSSP definition was simplified according to the following rules : H and G to helix, E and B to strand and all other states considered as coil, in agreement with PSIPRED definition [30].

The fraction of secondary structure (*SS*) is defined as:

$$SS=\frac{n_{ss}}{N}*100,$$

where *n*_*ss *_is the number of residues located in well-defined secondary structure elements, and N is the number of protein residues.

The secondary structure for each decoy was also compared with the corresponding secondary structure predicted by PSIPRED. Accordingly, the relative consensus secondary structure (*SSc*) was defined as the ratio:

$$SSc=\frac{n_{c}}{N}*100,$$

where *n*_*c *_is the number of residues located in corresponding secondary structure elements according to DSSP definition and PSIPRED secondary structure prediction.

Generally, native structures are characterized by hydrophobic residues clustered in buried regions. Therefore, the number of contacts between hydrophobic residues was chosen as a possible relevant parameter to discriminate among correct and incorrect models. According to our definition, a contact is present if the distance between two residues is greater than 2.5 Å and lower or equal to 5 Å [54]. Given *n *hydrophobic residues, the number of hydrophobic contacts (*Q*) is normalized relative to the number of all possible contacts:

$$rQ=\frac{2Q}{n(n-1)!},$$

Moreover, to keep into account the stereochemical quality of the model, some PROCHECK parameters were considered (Table 4).

**Table 4:** PROCHECK parameters. PROCHECK parameters used in AIDE. The G-factor, which is a log-odds score based on the observed distributions of stereochemical parameters, provides a measure of how "normal", or alternatively how "unusual", a given stereochemical property is.

Parameter
Percentage of residue in Ramachandran plot core regions
Percentage of residue in Ramachandran plot allowed regions
Percentage of residue in Ramachandran plot generously allowed regions
Percentage of residue in Ramachandran plot disallowed regions
Number of bad contacts
G-factor for dihedral angles
G-factor for covalent bonds
Overall G-factor

### Model accuracy measures

Quality of protein models was evaluated by means of five different descriptors, using the crystal structure as reference: RMSD on the backbone atoms, TM-score [33], GDT-TS [32], LG-score [32] and MaxSub [34]. RMSD was computed on the backbone atoms after superposing the model structure on the crystal structure, using the program CE [55].

TM-score was developed to evaluate the topology similarity of two protein structures [33]. TM-score values fall into the interval [0, 1]. Scores equal or below 0.17 indicate that the prediction has a reliability compared to a random selection from the PDB library.

GDT-TS gives an estimation of the largest number of residues that can be found in which all distances between the model and the reference structure are shorter than the cutoff D. The number of residues is measured as a percentage of the length of the target structure. The values of GDT-TS fall into the interval [0–1], with a GDT-TS of 1 corresponding to perfect superposition.

The LG-score represents the significance (P-value) of a score (S str [56]) associated to the best subpart of a structural alignment between the model and the correct structure. The value is measured by using a structural P-value ranging from 0 to 1, with a value of 0 corresponding to optimal superposition.

MaxSub is calculated from the largest number of residues that superimpose well over the reference structure, and produces a normalized score that ranges between 0 and 1. A MaxSub value of 1 is associated to perfect superposition.

### Neural network

Four layers feed forward neural networks were used, with fifteen neurons in the input layer, two neurons in two hidden layers and one neuron in the output layer. A linear activation function was chosen for all neurons.

For each accuracy measure chosen to evaluate proteins quality (RMSD, TM-score, GDT-TS, LG-score and MaxSub) a different neural network was trained.

The inverse of the Pearson correlation coefficient (CC) between the true and the predicted data was used as performance function.

$${\left\{CC\right\}}^{-1}={\left\{\frac{{\left(t-\mu_{t}\right)}^{T}(y-\mu_{y})}{(M-1)\sigma_{t}\sigma_{y}}\right\}}^{-1}$$

where ***t ***is the vector of predicted values for each decoy, ***y ***is the vector of true values, *μ*_*t*_, *σ*_*t*_, *μ*_*y*_, *σ*_*y *_are the averages and the standard deviations of predicted and true values, respectively, and *M *is the number of decoys.

Optimization of neural networks was carried out using the attractive-repulsive particle swarm optimization algorithm (AR-PSO) [37], which is a modification of the original PSO method [57,58]. PSO is a stochastic population-based optimization approach which explores the hyper-dimensional parameters space of a population of candidate solutions named particles. Particles fly over the solution space looking for the global optimum. Each particle retains an individual memory of the best position visited and a global memory of the best position visited by all the particles.

A particle calculates its next position combining information from its last movement, the individual memory, the global memory and a random component.

The PSO updating rule is described as follow:

$$\{\begin{array}{l} v_{i,t+1}=\mu v_{i,t}+c_{1}(w_{i,t}^{best}-w_{i,t})+c_{2}(w_{t}^{global}-w_{i,t})\\ w_{i,t+1}=w_{i,t}+v_{i,t+1} \end{array}$$

in which ***w***_*i*, *t*+1 _represents the position vector of the particle *i *at time *t *(i.e. the neural network weights), $w_{i,t}^{best}$ is the best position identified by the particle *i *so far (i.e. the neural network weights associated with the best performance value) and $w_{t}^{global}$ is the best position identified among all the particles. The vector ***v ***represents the particles velocity, which is computed as the difference between two positions and assuming unitary time.

The term ($w_{i,t}^{best}$ - ***w***_*i*, *t*_) represents the individual memory component and ($w_{t}^{global}$ - ***w***_*i*, *t*_) is the global one. These two terms are rescaled by the random coefficients *c*_1 _and *c*_2_, respectively. The *μ *coefficient is used to rescale the velocity.

Starting particle positions and velocities were initialized at random. To reduce the problem of premature convergence to relative minima, the Attractive-Repulsive modification has been introduced [37]. This modification defines a measure of global diversity (*D*) among the particles as:

$$D=\frac{1}{S}\sum_{i=1}^{S}\sqrt{\sum_{j=1}^{N}{\left(w_{ij}-{\bar{w}}_{j}\right)}^{2}}$$

where *S *is the number of particles in the swarm, *N *is space dimension (the number of networks weights) and ${\bar{w}}_{j}$ is the average of the parameter *j *among the particles.

If D falls below a minimal threshold (*t*_*min*_) the update rule is inverted as follow

$$\{\begin{array}{l} v_{i,t+1}=\mu v_{i,t}+(-1)c_{1}(w_{i,t}^{best}-w_{i,t})+(-1)c_{2}(w_{t}^{global}-w_{i,t})\\ w_{i,t+1}=w_{i,t}+v_{i,t+1} \end{array}$$

causing the particles to spread in the phase space. If D reaches a maximal threshold (*t*_*max*_) the update rule is restored as in the standard PSO method. We choose *t*_*min *_= 0.1 and *t*_*max *_= 5.0.

The parameters *c*1, *c*2 and *μ *were set as in the original PSO method as *c*1 = *c*2 ∈ [0.0, 2.0] and *μ *= 0.7298. The maximum number of iterations was set to 10000. A population size of 5 particles was chosen. It should be noted that standard training algorithms such as gradient descent back-propagation, Levenberg-Marquardt and BFGS, led to poorer results when compared to the particle swarm optimization (data not shown).

### Statistical analysis

The following statistical parameters were used: Pearson correlation coefficient, already described in the neural network section, fraction enrichment (F.E.) and *Z_nat*.

Fraction enrichment (F.E.) is defined as the fraction of the top 10% conformations featuring best structural resemblance to the native structure among the top 10% best scoring conformations.

*Z*_*nat *_is the Z-score of the X-ray structure compared to the ensemble of decoys structures. It is computed using the following equation:

$$Z_{nat}=\frac{score_{native}-\mu_{decoys}}{\sigma_{decoys}}$$

Higher *Z*_*nat *_values correspond to higher capacity to discriminate between the native structure and the corresponding decoys.

The Receiver Operating Characteristic (ROC) graph is a plot of all sensitivity/specificity pairs resulting from continuously varying the decision threshold over the range of results observed. The sensitivity or true positive fraction is reported on the y-axis, while the x-axis represents the 1-specificity or true negative fraction. A test with perfect discrimination (no overlap between the two distribution of results) has a plot curve that passes through the upper left corner, where both specificity and sensitivity are 1.00. The ipotetical plot of a test with no discrimination between the two groups is a 45° line going from the lower left to the upper right corner.

Qualitatively, the closer the plot is to the upper left corner, the higher the overall accuracy of the test.

## Authors' contributions

MP carried out the computational work. MP, PE and DGL carried out the data analysis. All authors contributed to the design of the study. All authors read and revised the manuscript.

## Availability and requirements

Project name: AIDE

Project home page: 

Operating system(s): Linux, Unix

Programming language: Fortran77/90, Perl

Licence: GNU GPLv3

Any restrictions to use by non-academics: No restrictions

## Supplementary Material

Additional file 1 Linear models. Linear models obtained with linear regression and M5-prime attribute selection algorithm. Click here for file

Additional file 2 Comparison of neural network performances. Comparison of neural network performances using different training algorithms. Click here for file

Additional file 3 Accuracy parameters loading plot. Loading plot of the accuracy parameters correlation matrix obtained by principal component analysis. Given the accuracy parameters matrix, where each row represents a different model and the columns are the descriptors used as quality measure (GDT_TS, LG-score, MaxSub, RMSD and TM-score), the correlation matrix (see additional file 5) was computed and analyzed by principal component analysis. Only the first two principal components are plotted. Click here for file

Additional file 4 Accuracy parameters correlation matrix. Pearson correlation matrix of predicted accuracy parameters for the test-set. Click here for file

Additional file 5 Manual assessment of CASP5 models vs TM-score. Manual assessment of different models of 13 targets of CASP5 belonging to the category of "new fold" and "fold recognition". Each model has been classified into one of the following three classes : "excellent", "good" and "bad", and showed in the figure as blue, green and red circles, respectively [42]. Each target is represented into a different panel, where the horizontal axes indicates the model number and the vertical axes is the TM-score. Click here for file

Additional file 6 Manual assessment of CASP5 models vs LG-score. Manual assessment of different models of 13 targets of CASP5 belonging to the category of "new fold" and "fold recognition". Each model has been classified into three classes : "excellent", "good" and "bad", and showed in the figure as blue, green and red circles, respectively [42]. Each target is represented into a different panel where the horizontal axes indicates the model number and the vertical axes is the LG-score. Click here for file

Additional file 7 Manual assessment of CASP5 models vs RMSD. Manual assessment of different models of 13 target of CASP5 belonging to the category of "new fold" and "fold recognition". Each model has been classified into three classes : "excellent", "good" and "bad" showed in the figure as blue, green and red circles, respectively [42]. Each target is represented into a different panel where the horizontal axes indicates the model number and the vertical axes is the RMSD. Click here for file

Additional file 8 ROC curves derived accuracy. Sensitivity and specificity of AIDE TM-score, AIDE RMSD and AIDE LG-score, as obtained from the ROC curves at the chosen threshold. Click here for file
