# Supplementary material for: Validation of protein models by a neural network approach
Source: BMC Bioinformatics. 2008 Jan 29;9:66. doi: 10.1186/1471-2105-9-66 (PMC2276493; doi:10.1186/1471-2105-9-66)
Supplement: Additional file 1 — Linear models. Linear models obtained with linear regression and M5-prime attribute selection algorithm. [file 1471-2105-9-66-S1.pdf]

Linear models obtained with linear regression and M5-prime attribute selection algorithm.

For each accuracy measure the models weights, the excluded parameters and the prediction performance are shown. The training-set and and the test-set used for obtaining and evaluating the linear model are the same used for the neural network.

Parameters abbreviations	
SS	Fraction of secondary structure
CORE	Percentage of residue in Ramachandran plot CORE
ALL	Percentage of residue in Ramachandran plot allowed regions
GENALL	Percentage of residue in Ramachandran plot generously allowed regions
DISALL	Percentage of residue in Ramachandran plot disallowed regions
nBC	Number of bad contacts
DH_G	G-factor for dihedral angles
COV_B	G-factor for covalent bonds
$OVER_G$	Overall G-factor
HB_{SAS}	hydrophobic relative solvent accessible surface
HY_{SAS}	hydrophilic relative solvent accessible surface
SSc	Secondary structure consensus
rQ	Relative number of hydrophobic contacts
L	Number of residues

1 RMSD

$1.9305SS - 0.2326DH_G - 0.351COV_G + 0.9225OVER_G - 0.1815CORE - 0.1591ALL - 0.1899GENALL - 0.1215DISALL - 4.1309HB_{SAS} - 12.1232HY_{SAS} - 0.0362SSc - 0.0382rQ - 0.0022L + 30.4336$

Excluded parameters : nBC

Pearson correlation coefficient : 0.30

2 GDT_TS

$-0.3586SS - 0.0006nBC - 0.0434DG_G - 0.036COV_G + 0.0463OVER_G - 0.0018ALL + 0.0041DISALL - 1.5662HB_{SAS} + 0.0025SSc - 0.0088rQ - 0.0001L + 1.2403$

Excluded parameters : CORE, GENALL, HY_{SAS}

Pearson correlation coefficient : 0.32

3 TM-score

$-0.3937SS - 0.0006BC - 0.0369DG_G - 0.0168COV_G - 0.002ALL + 0.0026DISALL - 1.6329HB_{SAS} + 0.0027SSc - 0.0163rQ + 0.0003L + 1.2718$

Excluded parameters : CORE, GENALL, HY_{SAS}, $OVER_G$

Pearson correlation coefficient : 0.31

4 MaxSub

$-0.3639SS + -0.0006nBC + -0.025DG_G + -0.0239COV_G + 0.0188OVER_G + -0.0016ALL +$
$0.0029DISALL + -1.3149HB_{SAS} + 0.1461HY_{SAS} + 0.0024SSc + -0.005rQ + 0.9618$

Excluded parameters : CORE, GENALL, L

Pearson correlation coefficient : 0.33

5 LG-score

$1.0516SS + 0.0473DG_G + 0.1107COV_G + -0.2139OVER_G + 0.0014ALL + -0.0161DISALL +$
$-0.8566HB_{SAS} + -1.0837HY_{SAS} + -0.0015rQ + 0.0328SSc + -0.0008L + 1.2131$

Excluded parameters : nBC, CORE, GENALL, GENALL, L

Pearson correlation coefficient : 0.28
