# Supplementary material for: Validation of protein models by a neural network approach
Source: BMC Bioinformatics. 2008 Jan 29;9:66. doi: 10.1186/1471-2105-9-66 (PMC2276493; doi:10.1186/1471-2105-9-66)
Supplement: Additional file 2 — Comparison of neural network performances. Comparison of neural network performances using different training algorithms. [file 1471-2105-9-66-S2.pdf]

Comparison of neural network performances

For each assessment measure (GDT_TS, LG-score, MaxSub, RMSD and TM-score) the same neural network was trained using different algorithms : Levenberg-Marquardt(LM), Gradient Descent(GD) and Particle Swarm Optimzation(PSO).

Gradient descent parameters :

Training epochs : 100

Minimum performance gradient : 10^{-10}

Learning rate : 0.01

Levenberg-Marquardt parameters :

Training epochs : 100

Minimum performance gradient : 10^{-10}

μ : 10^{-3}

μ increment : 10

μ decrement : 0.1

PSO:

See section Neural Networks in Methods

The performance of the neural networks was computed as Pearson correlation coefficient on the overall test-set.

	LM	GD	PSO
GDT_TS	0.36	0.38	0.45
LG-score	0.35	0.48	0.51
MaxSub	0.49	0.39	0.51
RMSD	0.38	0.33	0.42
TM-score	0.43	0.49	0.49
