# Supplementary material for: Validation of protein models by a neural network approach
Source: BMC Bioinformatics. 2008 Jan 29;9:66. doi: 10.1186/1471-2105-9-66 (PMC2276493; doi:10.1186/1471-2105-9-66)
Supplement: Additional file 3 — Accuracy parameters loading plot. Loading plot of the accuracy parameters correlation matrix obtained by principal component analysis. Given the accuracy parameters matrix, where each row represents a different model and the columns are the descriptors used as quality measure (GDT_TS, LG-score, MaxSub, RMSD and TM-score), the correlation matrix (see additional file 5) was computed and analyzed by principal component analysis. Only the first two principal components are plotted. [file 1471-2105-9-66-S3.PDF]

Accuracy parameters correlation matrix

Pearson correlation matrix of predicted accuracy parameters for the test-set.

	GDT_TS	LG-score	MaxSub	RMSD	TM-score
GDT_TS	1.00	-0.77	-0.98	-0.76	0.95
LG-score	-0.77	1.00	0.71	0.68	-0.79
MaxSub	-0.98	0.71	1.00	0.78	-0.93
RMSD	-0.76	0.68	0.78	1.00	-0.73
TM-score	0.95	-0.79	-0.93	-0.73	1.00
