# Supplementary material for: Validation of protein models by a neural network approach
Source: BMC Bioinformatics. 2008 Jan 29;9:66. doi: 10.1186/1471-2105-9-66 (PMC2276493; doi:10.1186/1471-2105-9-66)
Supplement: Additional file 4 — Accuracy parameters correlation matrix. Pearson correlation matrix of predicted accuracy parameters for the test-set. [file 1471-2105-9-66-S4.pdf]

ROC curves derived accuracy

Sensitivity and specificity of AIDE TM-score, AIDE RMSD and AIDE LG-score as obtained from the ROC curves at the chosen threshold.

	TM-score	RMSD	LG-score
Threshold	0.31	4.96Å	0.35
Sensitivity	40%	90%	88%
Specificity	96%	70%	74%
