# Supplementary figures and images for: Validation of protein models by a neural network approach
Source: BMC Bioinformatics. 2008 Jan 29;9:66. doi: 10.1186/1471-2105-9-66 (PMC2276493; doi:10.1186/1471-2105-9-66)

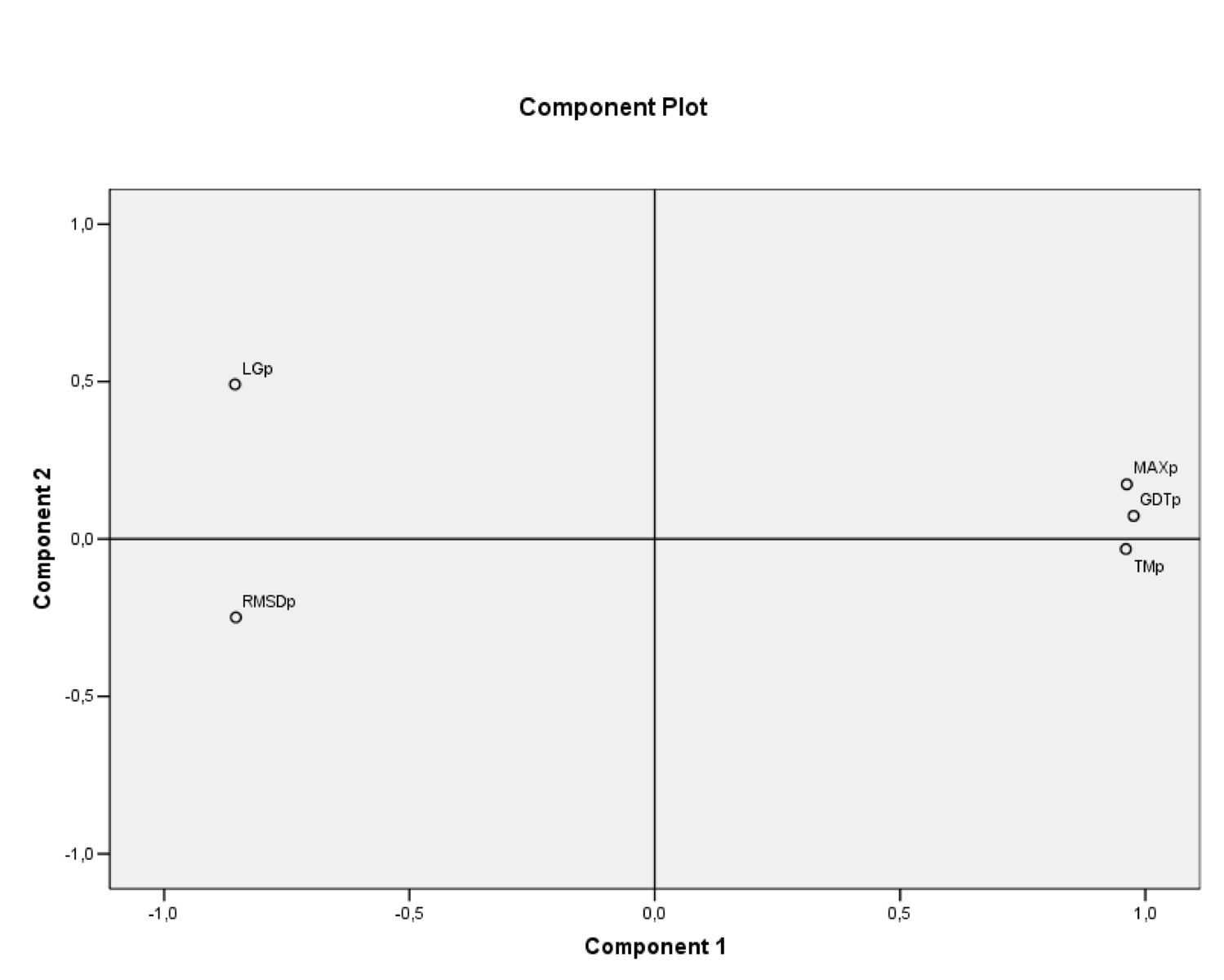

Supplement: Additional file 5 — Manual assessment of CASP5 models vs TM-score. Manual assessment of different models of 13 targets of CASP5 belonging to the category of "new fold" and "fold recognition". Each model has been classified into one of the following three classes : "excellent", "good" and "bad", and showed in the figure as blue, green and red circles, respectively [42]. Each target is represented into a different panel, where the horizontal axes indicates the model number and the vertical axes is the TM-score. [file 1471-2105-9-66-S5.png]

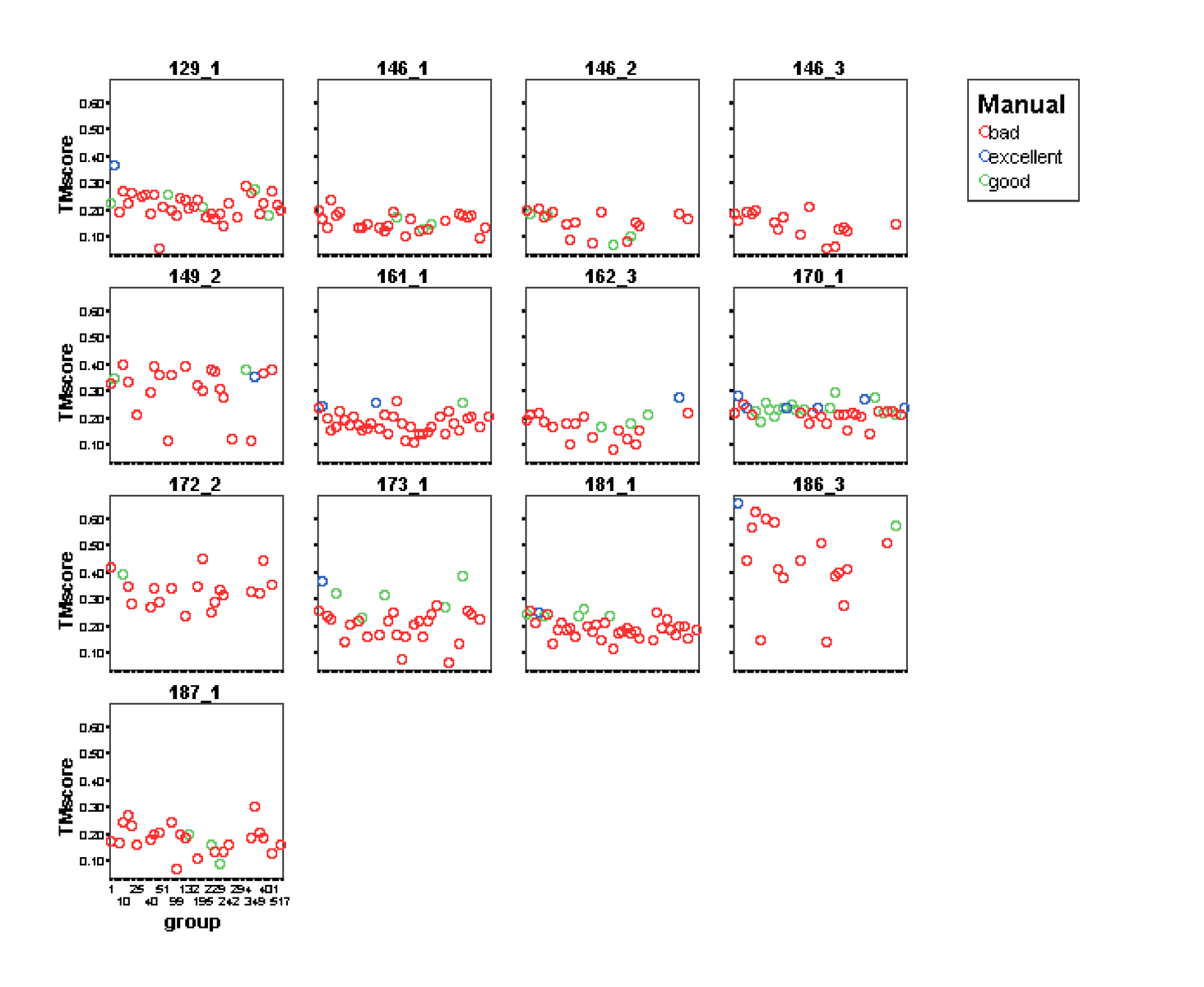

Supplement: Additional file 6 — Manual assessment of CASP5 models vs LG-score. Manual assessment of different models of 13 targets of CASP5 belonging to the category of "new fold" and "fold recognition". Each model has been classified into three classes : "excellent", "good" and "bad", and showed in the figure as blue, green and red circles, respectively [42]. Each target is represented into a different panel where the horizontal axes indicates the model number and the vertical axes is the LG-score. [file 1471-2105-9-66-S6.png]

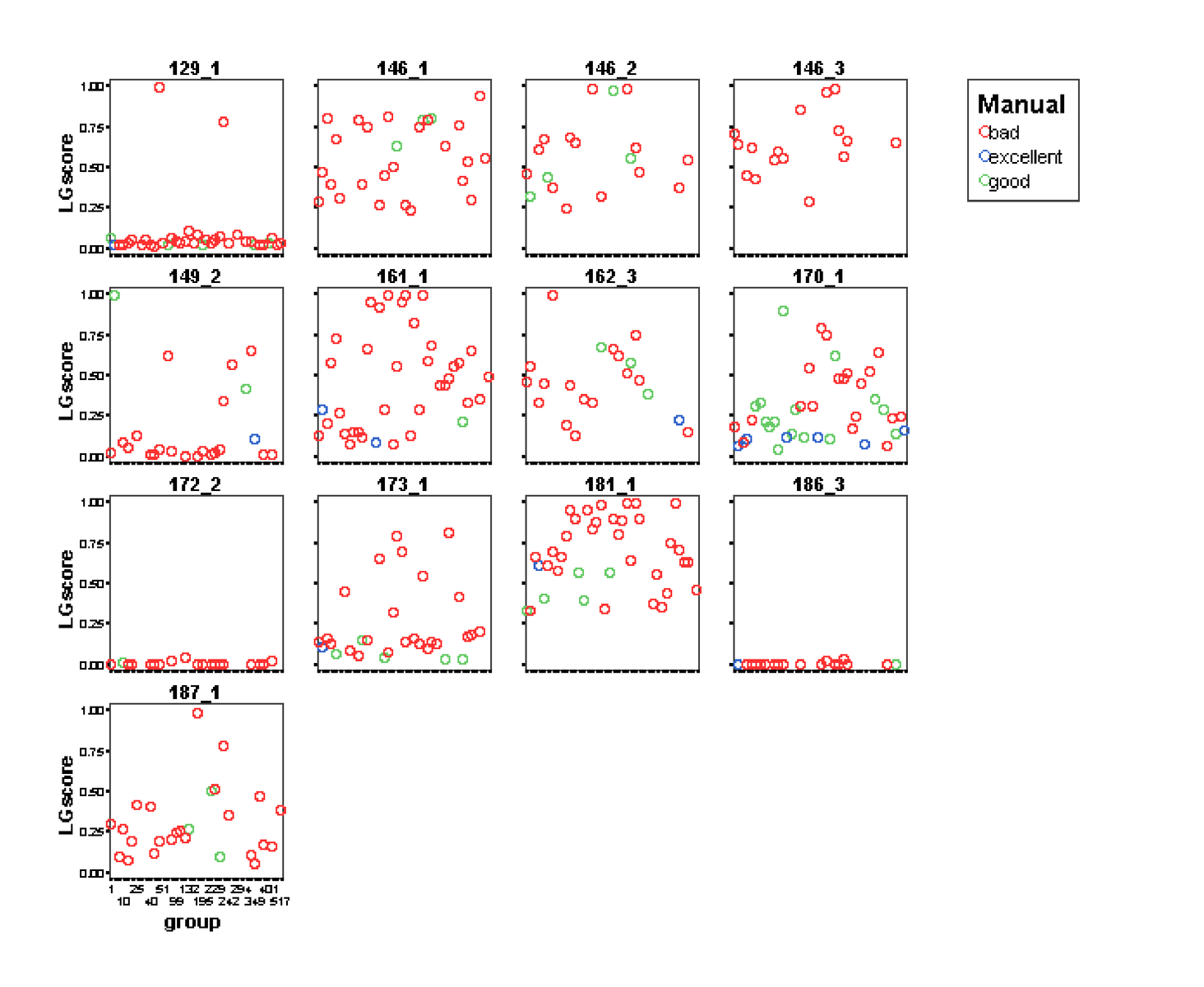

Supplement: Additional file 7 — Manual assessment of CASP5 models vs RMSD. Manual assessment of different models of 13 target of CASP5 belonging to the category of "new fold" and "fold recognition". Each model has been classified into three classes : "excellent", "good" and "bad" showed in the figure as blue, green and red circles, respectively [42]. Each target is represented into a different panel where the horizontal axes indicates the model number and the vertical axes is the RMSD. [file 1471-2105-9-66-S7.png]

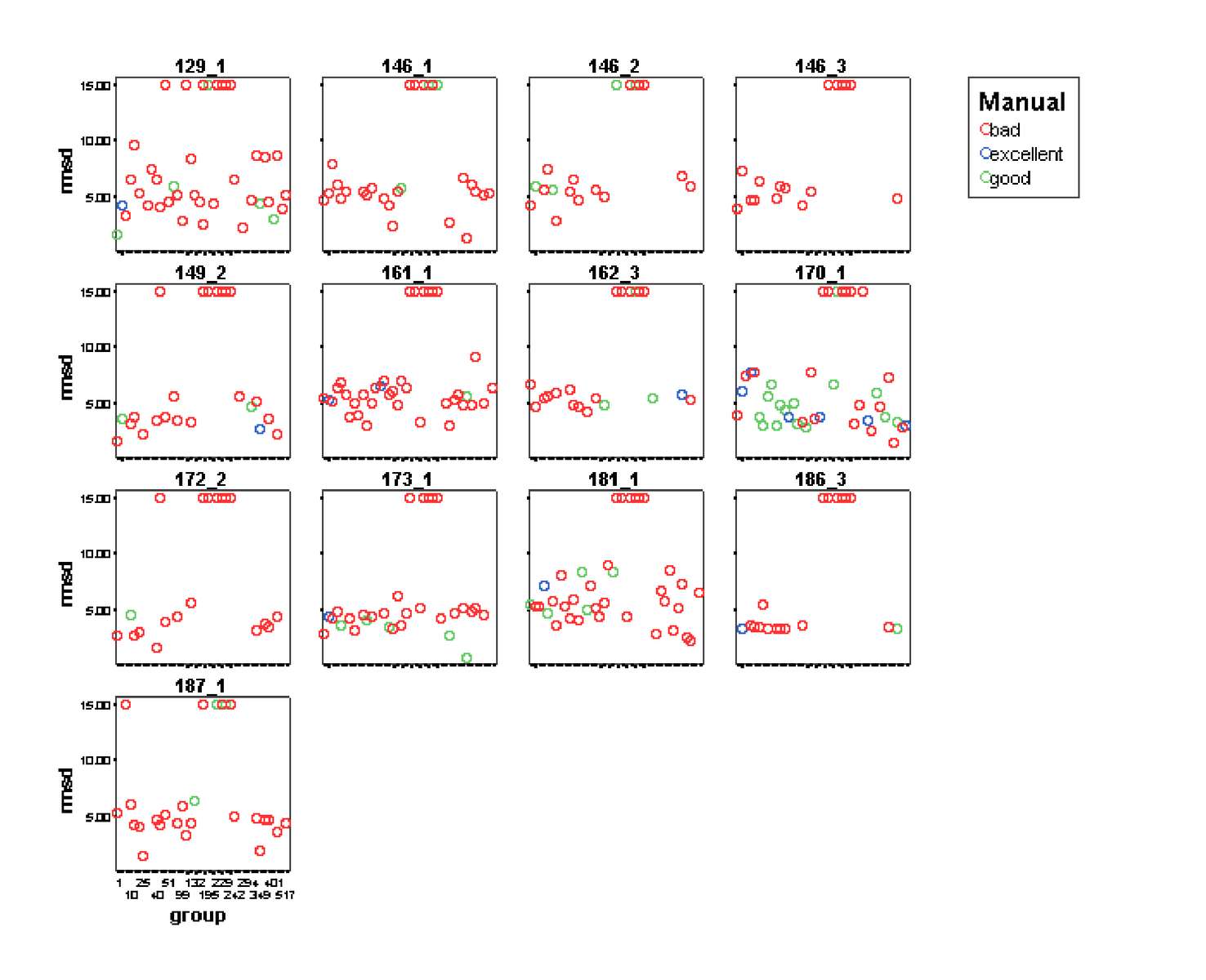

Supplement: Additional file 8 — ROC curves derived accuracy. Sensitivity and specificity of AIDE TM-score, AIDE RMSD and AIDE LG-score, as obtained from the ROC curves at the chosen threshold. [file 1471-2105-9-66-S8.PNG]
